# Whole-blood culture-derived cytokine combinations for the diagnosis of tuberculosis

**DOI:** 10.3389/fimmu.2024.1397941

**Published:** 2024-06-12

**Authors:** Anne Ahrens Østergaard, Søren Feddersen, Mike B. Barnkob, Rasmus Bank Lynggaard, Amanda Cecilie Annie Karstoft, Maria Borup, Ingrid Louise Titlestad, Torben Tranborg Jensen, Ole Hilberg, Christian Wejse, Stephanie Bjerrum, Morten Blaabjerg, Kristian Assing, Isik Somuncu Johansen

**Affiliations:** ^1^ Research Unit of Infectious Diseases, Department of Clinical Research, University of Southern Denmark, Odense, Denmark; ^2^ Department of Infectious Diseases, Odense University Hospital, Odense, Denmark; ^3^ Department of Clinical Biochemistry, Odense University Hospital, Odense, Denmark; ^4^ Clinical Biochemistry, Department of Clinical Research, University of Southern Denmark, Odense, Denmark; ^5^ Department of Clinical Immunology, Odense University Hospital, Odense, Denmark; ^6^ Department of Respiratory Medicine, Odense University Hospital, Odense, Denmark; ^7^ Odense Respiratory Research Unit (ODIN), Department of Clinical Research, University of Southern Denmark, Odense, Denmark; ^8^ Department for Pulmonary Diseases, Esbjerg Hospital, Esbjerg, Denmark; ^9^ Department of Medicine, Vejle Hospital, Hospital Lillebælt, Vejle, Denmark; ^10^ Department of Infectious Diseases, Aarhus University Hospital, Aarhus, Denmark; ^11^ Center for Global Health (GloHAU), Department of Public Health, Aarhus University, Aarhus, Denmark; ^12^ Department of Infectious Diseases, University Hospital of Copenhagen, Rigshospitalet, Copenhagen, Denmark; ^13^ Research Unit of Neurology, Department of Clinical Research, University of Southern Denmark, Odense, Denmark; ^14^ Department of Neurology, Odense University Hospital, Odense, Denmark; ^15^ Research Unit of Clinical Immunology, Department of Clinical Research, Odense, Denmark

**Keywords:** tuberculosis, tuberculosis infection, recursive feature elimination (RFE), random forest model (RF), conditions mimicking tuberculosis, whole blood stimulation, cytokines, diagnostic test

## Abstract

**Introduction:**

The diagnosis of tuberculosis (TB) disease and TB infection (TBI) remains a challenge, and there is a need for non-invasive and blood-based methods to differentiate TB from conditions mimicking TB (CMTB), TBI, and healthy controls (HC). We aimed to determine whether combination of cytokines and established biomarkers could discriminate between 1) TB and CMTB 2) TB and TBI 3) TBI and HC.

**Methods:**

We used hemoglobin, total white blood cell count, neutrophils, monocytes, C-reactive protein, and ten Meso Scale Discovery analyzed cytokines (interleukin (IL)-1β, IL-2, IL-4, IL-6, IL-8, IL-10, IL-12p70, IL-13, interferon (IFN)-ɣ, and tumor necrosis factor (TNF)-α) in TruCulture whole blood tubes stimulated by lipopolysaccharides (LPS), zymosan (ZYM), anti-CD3/28 (CD3), and unstimulated (Null) to develop three index tests able to differentiate TB from CMTB and TBI, and TBI from HC.

**Results:**

In 52 persons with CMTB (n=9), TB (n=23), TBI (n=10), and HC (n=10), a combination of cytokines (LPS-IFN-ɣ, ZYM-IFN-ɣ, ZYM-TNF-α, ZYM-IL-1β, LPS-IL-4, and ZYM-IL-6) and neutrophil count could differentiate TB from CMTB with a sensitivity of 52.2% (95% CI: 30.9%–73.4%) and a specificity of 100 % (66.4%-100%). Null- IFN-ɣ, Null-IL-8, CD3-IL-6, CD3-IL-8, CD3-IL-13, and ZYM IL-1b discriminated TB from TBI with a sensitivity of 73.9% (56.5% - 91.3%) and a specificity of 100% (69.2-100). Cytokines and established biomarkers failed to differentiate TBI from HC with ≥ 98% specificity.

**Discussion:**

Selected cytokines may serve as blood-based add-on tests to detect TB in a low-endemic setting, although these results need to be validated.

## Introduction

Tuberculosis (TB) is caused by the *Mycobacterium tuberculosis* (*Mtb*) complex. TB is a global health problem, with an estimated 10.6 million new TB cases in 2022 (1). Despite recent molecular developments, diagnosis of TB remains challenging, with one-third of persons with TB globally remaining undiagnosed in 2022 (1). Moreover, about a quarter of the world’s population is estimated to have TB infection (TBI), a persistent immune response to stimulation by *Mtb* antigens without evidence of TB disease. TBI is essential to the TB elimination strategy (2). However, no specific standard for diagnosing TBI exists. The development of accurate diagnostic tools is necessary and crucial to distinguish between the host immune responses in TBI and TB (3).

TB primarily affects the lungs as pulmonary TB (PTB), and the diagnosis relies on the culture of *Mtb*, which is considered the gold standard. However, in 2021, only 72.0% of persons diagnosed with TB in the European region received microbiological confirmation, with PTB confirmed in 86.2% of cases (4). Laboratory confirmation of extrapulmonary TB (EPTB) was low even in high-resource settings due to the overlap of TB symptoms with those of other diseases (5, 6), referred to as conditions mimicking TB (CMTB). Further, difficulties in obtaining relevant material and low bacterial load of TB result in suboptimal sensitivity of current diagnostic tools (7). Consequently, new biomarkers to diagnose TB should be less strongly associated with the bacillary load.

The interferon gamma release assays (IGRA) and tuberculin skin test (TST) in Bacillus Calmette–Guérin (BCG) unvaccinated persons can detect immune reactivity toward *Mtb* with ≥97% specificity (8, 9). However, it is believed that *Mtb* can be cleared by the innate immune response without a detectable persisting immune response using IGRAs or TSTs and by the acquired immune response with the persisting immune response (10). Currently, there are no tests available to detect this.

Host-based biomarkers have greater potential than pathogen-based biomarkers for diagnosing TB (11). The World Health Organization (WHO) has thus desired non-sputum-based biomarker tests for TB with high specificity (target 98%) and a sensitivity of at least 65% in all groups, including EPTB (12). The host’s response to *Mtb* is complex and eludes complete characterization (13), but multiple different cytokines, such as interleukin (IL)-4, IL-10, IL-13 (14), interferon (IFN)-γ, IL-12p70 (15), IL-6 (16), IL-8 (17), tumor necrosis factor (TNF)-α (18), IL-1β (19), and IL-2 (20) have been implicated in immune control of *Mtb.*

However, no single cytokine measurements fulfill the WHO criteria for distinguishing TB from TBI and CMTB (11). Also, no single cytokine biomarker can differentiate between TB and TBI (21). Cytokine levels have successfully been explored to discriminate between PTB and CMTB (22). Thus, better biomarkers distinguishing between the host immune responses in TBI and TB, including EPTB, are needed.

Both innate and adaptive immunity is crucial for *Mtb* immunity (23, 24). Toll-like receptor (TLR) 2, by forming heterodimers with TLR1 and TLR6, can recognize a range of mycobacterial products (25). TLR4 also recognizes *Mtb-*derived proteins (mammalian cell entry proteins), lipoproteins, and glycolipids (26). While T-cell immunity toward *Mtb* may display retarded kinetics (27), T-cell immunity is nevertheless critical for protective immune responses to *Mtb* (28).

This explorative study aims to determine whether T-cell (CD3- and CD28-stimulated T cells)- and innate immunity (TLR2 and TLR4 induced)-derived cytokines and established biomarkers [hemoglobin, C-reactive protein (CRP), total white blood cell count (WBC), neutrophils, and monocytes] may delineate useful biomarkers to discrimination between 1) TB from CMTB, 2) TB from TBI, and 3) TBI from healthy controls (HC) with a ≥98% specificity as an add-on test to current TB diagnostics.

## Method

### Study design and participants

In this prospective study, we consecutively included adult (≥18 years) patients, after written informed consent, with symptoms and signs of TB or TBI before initiating antituberculous treatment. We defined symptoms and signs of TB as systemic disease with at least 2 weeks of either fever, weight loss, night sweats, fatigue, chest pain, or cough; laboratory or radiological findings suggestive of TB; or clinical suspicion leading to treatment by the treating physician. TBI was defined as persons with a positive IGRA test reported by the laboratory according to the manufacturer’s instructions (29), no previous TB-preventive therapy without renewed TB exposure, and absence of TB disease evaluated by the treating physician. Participants were recruited from respiratory medicine and infectious diseases departments and outpatient clinics from four secondary and tertiary Danish hospitals (Odense, Esbjerg, Aarhus, and Hillerød) from September 2020 to April 2022. We included HC with a negative IGRA test, no diabetes, pregnancy, immunosuppressive disorder, and no fever or malaise at inclusion as controls among the staff at the Department of Infectious Diseases, Odense University Hospital.

We classified TB diagnosis as follows: Definite TB was verified by culture isolation of *Mtb*, positive specific PCR, or acid-fast bacilli by microscopy. Probable TB was diagnosed based on symptoms or radiological or histological findings compatible with TB. Possible TB was diagnosed based on clinical suspicion and response to antituberculous treatment. CMTB refers to cases of no laboratory findings of TB and confirmation of another disease, leading to a determination of antituberculous treatment or no initiation. After 2 months, we conducted a follow-up on patients exhibiting symptoms and signs of TB according to group patients in TB or CMTB.

We included both EPTB and PTB. In the case of both PTB and EPTB, we classified patients as PTB. Immunosuppressive disorders, including HIV, were not exclusion criteria.

We categorized the TB incidence rate in the country of birth (Low <10 TB cases/100,000 population, Medium; 10–40/100,000, High; >40/100,000) according to the WHO in 2019 (30). We obtained information on sex from the unique 10-digit personal identifier (CPR number) assigned to all residents at birth or after residing legally in Denmark for 3 months.

### Ethics

We conducted the study according to the Helsinki Declaration. The Danish Data Protection Agency (Jr: 18/42213 and Jr: 20/45850) and the Danish National Committee of Health Research Ethics (S-20180093) approved the study.

### Sample collection

We collected 1 ml of whole blood from all participants directly into four TruCulture® Whole Blood Collection tubes (Myriad/Rule Based Medicine, Austin, USA) containing immune cell stimulations: lipopolysaccharides (LPS) for TLR 4 stimulation, zymosan (ZYM) for TLR2 stimulation, anti-CD3 and anti-CD28 (CD3) for T-cell stimulation, and unstimulated (Null). Sampler creates a negative pressure by the plunger upon collection, which ensures an equal amount of whole blood in each tube. TruCulture tubes were stored at −20°C and heated to room temperature before blood collection. After blood filling, the tubes were incubated in a dry incubator at 37°C for 22 h ± 15 min. The liquid supernatants were transferred to other tubes and stored at −70°C until analysis. We included hemoglobin, WBC, neutrophils, monocytes, and CRP results within 16 h of whole-blood collection in the TruCulture tubes.

### Cytokine analysis

We analyzed the cytokines (IL-1β, IL-2, IL-4, IL-6, IL-8, IL-10, IL-12p70, IL-13, IFN-γ, and TNF-α) using the chemiluminescence-based Meso Scale Discovery platform (Meso Scale Diagnostics, Maryland, USA) and V-PLEX Plus Pro-inflammatory Panel 1 Human Kit (Meso Scale Diagnostics, Maryland, USA) per manufacturer’s instructions and reported in pg/ml. Further detail on the analysis can be found in the **Supplementary Material**.

### Cut-offs and test positivity

We chose cut-offs for all biomarkers according to a minimum of 98% specificity criteria by tabulating sensitivity against specificity to discriminate between groups for each biomarker. We defined the index test as positive if all included terms were fulfilled and negative if none or only some included terms were fulfilled.

### Statistics

We calculated medians with interquartile ranges (IQR) of age, established biochemical markers, and chemokines for all groups and compared medians using Mann–Whitney *U*-test as data were non-parametrically distributed. We used Scikit-learn (31), Matplotlib (32), and Stata 18.0/BE (StataCorp LLC, Texas, USA) for graphics and analyses.

We utilized k-nearest neighbors’ imputation (outcome category, sex, and age) to handle missing outcome variables in the dataset. Afterward, we applied recursive feature elimination using a random forest model to identify the most potent biomarkers. Recursive feature elimination is a variable selection technique that chooses the essential variables (biomarkers) of most importance in a larger dataset to develop a predictive model by removing the weakest features, allowing for the correlation of variables (33). We did not adjust for multiple testing as the method is non-p-value driven. We used min–max scaling (rescaling) to normalize hemoglobin for one analysis in males and females. We calculated bootstrapped 95% confidence intervals (CI) for values different from 1, exact binominal 95% CIs for values equal to 1, and illustrated the index tests performances by ROC (receiver operating characteristic) curves.

## Results

### Participant characteristics

**Figure 1:**
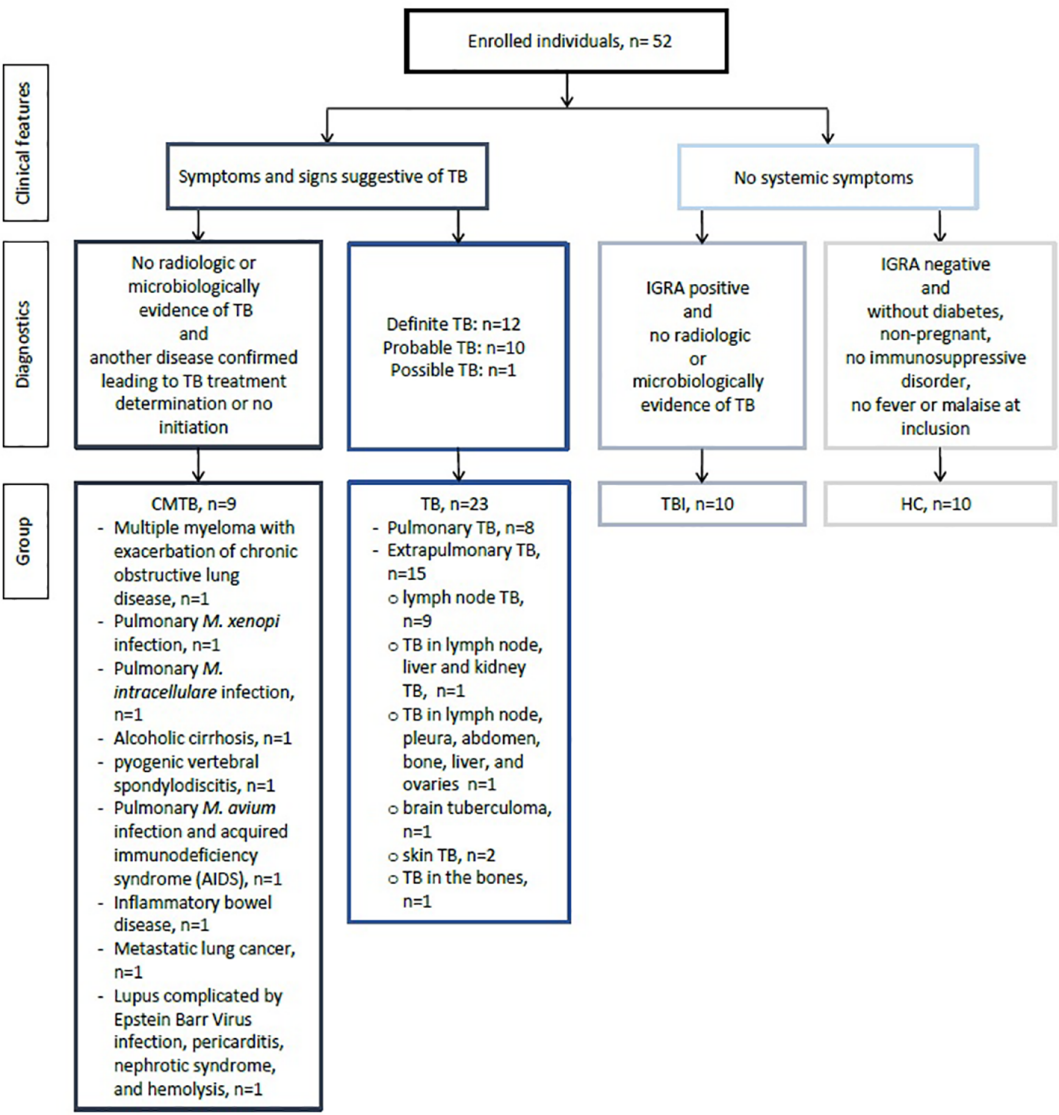
Flow chart of the study population. CMTB, conditions mimicking tuberculosis disease; HC, healthy controls; TB, tuberculosis; TBI, tuberculosis infection; IGRA, interferon gamma release assays.

We included 52 persons from four different hospitals in Denmark and divided them into four groups: TB, TBI, CMTB, and HC (**Figure 1**). The CMTB group consisted of malignant disease (n = 2), non-tuberculosis mycobacteria (n = 3), inflammatory bowel and rheumatic diseases (n = 2), alcoholic liver cirrhosis (n = 1), and pyogenic vertebral spondylodiscitis (n = 1). Persons with TBI (n = 10) reported new and unknown/older exposure. Of HC (n = 10), most reported *Mtb* exposure due to working in a healthcare setting. Participants had a median age (IQR) of 46.2 (36.5–59.5) years, 27 (51.9%) were female, 29 (59.2%) were born in Denmark, and 19/52 (38.8%) were born in a country with a high TB incidence rate (>40/100.000), including 15/23 (65.2%) of TB patients (**Table 1**). IGRA test results were available for 48/52 (92.3%) study participants. No IGRA tests were performed in four of the 23 TB patients, and the IGRA was negative in one TB patient. TB diagnosis was definite in 12 patients (PTB, n = 6; EPTB, n = 6), probable in 10 (PTB, n = 2; EPTB, n = 8), and possible in one EPTB.

**Table 1:** Study population characteristics.

Characteristics	CMTB	TB	TBI	HC	Total
**n, %**	9 (17.3)	23 (44.2)	10 (19.2)	10 (19.2)	52 (100)
**Median age in years** **[IQR]**	52.9 [46.5–62.5]	47.8 [31.2–61.7]	45.2 [37.9–56.6]	41.6 [35.0–53.4]	46.2 [36.5–59.5]
**Sex, female (%)**	3 (33.3)	12.0 (52.2)	6 (60.0)	6 (60.0)	27 (51.9)
**Birthplace in Denmark, n (%)**	6 (75.0)	7.0 (30.4)	6 (75.0)	10 (100)	29 (59.2)
TB incidence rate in birth country, n (%)					
Low (<10/100,000)	6 (75.0)	7.0 (30.4)	6 (75.0)	10 (100)	29 (59.2)
Medium (10–40/100,000)	0 (0.0)	1.0 (4.3)	0 (0.0)	0 (0.0)	1 (2.0)
High (>40/100,000)	2 (25.0)	15.0 (65.2)	2 (25.0)	0 (0.0)	19 (38.8)
IGRA result, n (%)					
Negative	8 (88.9)	1 (4.3)	0 (0.0)	10 (100)	19 (36.5)
Positive	1 (11.1)	18 (78.3)	10 (100)	0 (0.0)	29 (55.8)
Unknown	0 (0.0)	4 (17.4)	0 (0.0)	0 (0.0)	4 (7.7)
TB diagnosis, n (%)					
Definite	NA	12 (52.2)	NA	NA	12 (23.1)
Probable	NA	10 (43.5)	NA	NA	10 (19.2)
Possible	NA	1 (4.3)	NA	NA	1 (1.9)

Characteristics of persons with conditions mimicking tuberculosis (CMTB), tuberculosis (TB), tuberculosis infection (TBI), and healthy controls (HC). IGRA, interferon gamma release assays; IQR, interquartile range; NA, not applicable. TB incidence rate in birth country according to the World Health Organization, 2019.

### Biomarker performance

**Figure 2:**
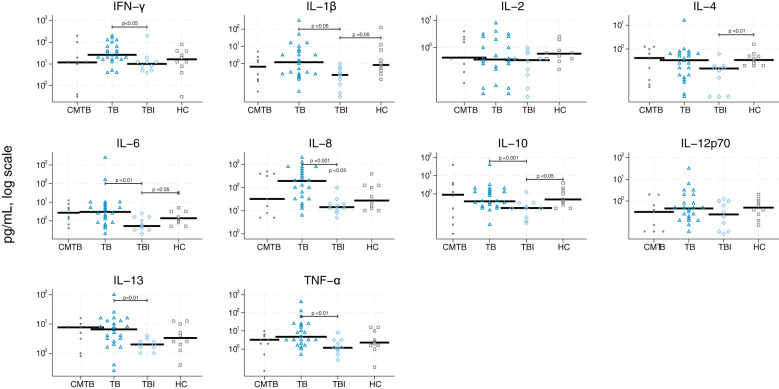
Scatter plot of cytokines derived from null stimulation. Scatter plot of cytokines derived from null stimulation in conditions mimicking tuberculosis (circle), tuberculosis (triangle), tuberculosis infection (rhomb), and healthy controls (square). Median concentrations are indicated by horizontal fat black bars. Mann–Whitney U-test indicates statistically significant differences between the groups indicated by thin black lines and the p-values. CMTB, condition-mimicking tuberculosis; HC, healthy controls. IFN-γ, interferon gamma; IL, interleukin; TNF-α, tumor necrosis factor α; TB, tuberculosis; TBI, tuberculosis infection.

**Figure 3:**
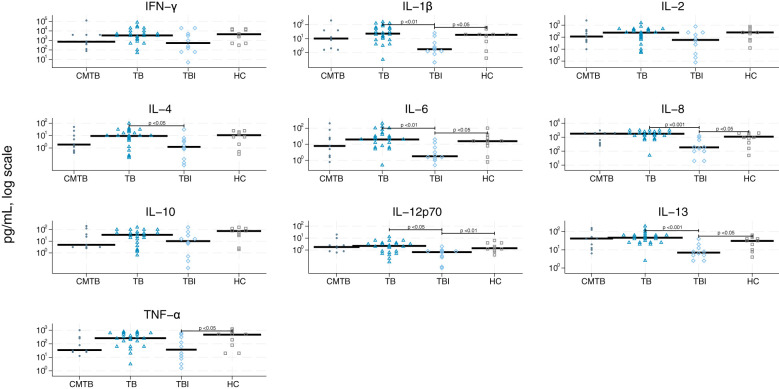
Scatter plot of cytokines derived from stimulation by anti-cluster of differentiation (CD) 3/CD28. Scatter plot of cytokines derived from stimulation by CD3/CD28 in conditions mimicking tuberculosis (circle), tuberculosis (triangle), tuberculosis infection (rhomb), and healthy controls (square). Median concentrations are indicated by horizontal fat black bars. Mann–Whitney U-test indicates statistically significant differences between the groups indicated by thin black lines and the p-values. CMTB, condition-mimicking tuberculosis; HC, healthy controls. IFN-γ, interferon gamma; IL, interleukin; TNF-α, tumor necrosis factor α; TB, tuberculosis; TBI, tuberculosis infection.

**Figure 4:**
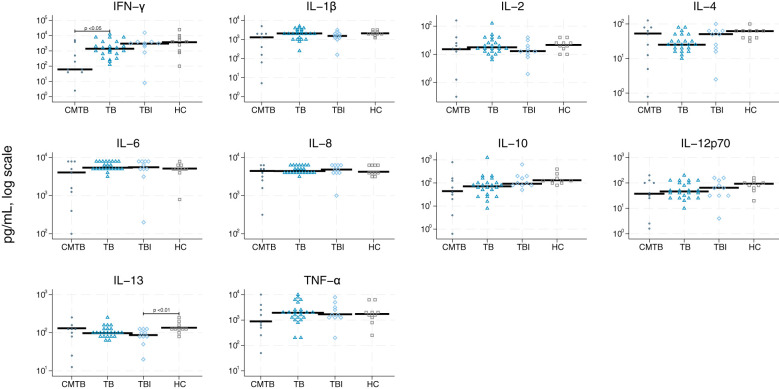
Scatter plot of cytokines derived from lipopolysaccharides. Scatter plot of cytokines derived from lipopolysaccharides (TLR2) in conditions mimicking tuberculosis (circle), tuberculosis (triangle), tuberculosis infection (rhomb), and healthy controls (square). Median concentrations are indicated by horizontal fat black bars. Mann–Whitney U-test indicates statistically significant differences between the groups indicated by thin black lines and the p-values. CMTB, condition-mimicking tuberculosis; HC, healthy controls. IFN-γ, interferon gamma; IL, interleukin; TNF-α, tumor necrosis factor α; TB, tuberculosis; TBI, tuberculosis infection.

**Figure 5:**
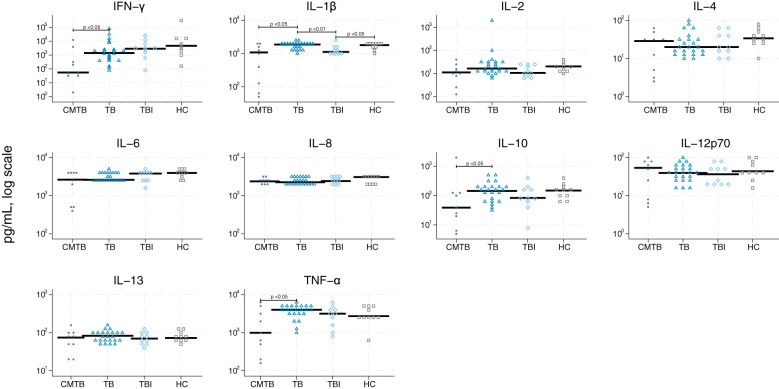
Scatter plot of cytokines derived from zymosan. Scatter plot of cytokines derived from stimulation by zymosan (TLR4) in conditions mimicking tuberculosis (circle), tuberculosis (triangle), tuberculosis infection (rhomb), and healthy controls (square). Median concentrations are indicated by horizontal fat black bars. Mann–Whitney U-test indicates statistically significant differences between the groups indicated by thin black lines and the p-values. CMTB, condition-mimicking tuberculosis; HC, healthy controls. IFN-γ, interferon gamma; IL, interleukin; TNF-α, tumor necrosis factor α; TB, tuberculosis; TBI, tuberculosis infection.

We graphically accessed the data for inter-analysis variance and found that the differences were very limited (**Supplementary Figure S1**). The single biomarker performance to differentiate between the four study groups is shown in **Table 2** and **Figures 2**–**5**. The median value of CRP (IQR) was significantly higher in persons with TB versus TBI (p < 0.05). The min–max scaled levels of hemoglobin were significantly lower in persons with CMTB compared to TB (p < 0.05) and lower in the TB group than in the TBI group (p < 0.05). Compared to TB patients, WBC and neutrophil counts were elevated in CMTB (p < 0.05). We found no difference in the level of established biochemical markers nor in cytokine concentrations between definite, probable, and possible TB nor PTB and EPTB (data not shown). Data without imputed values are shown in **Supplementary Table S2**.

**Table 2:** Biomarkers in conditions mimicking tuberculosis, tuberculosis, tuberculosis infection, and healthy controls.

Variables		CMTB	TB	TBI	HC	Significant differences in groups, p-value level by Mann–Whitney *U*-test		
**n (%)**		9 (17.3)	23 (44.2)	10 (19.2)	10 (19.2)	**TB vs. CMTB**	**TB vs TBI**	**TBI vs. HC**
**CRP mg/L [IQR]** Ref:<5.0		47.0 [11.0–59.0]	7.1 [2.5–23.0]	0.9 [0.3–2.0]	0.8 [0.6–1.4]		<0.05	
Hemoglobin mmol/L [IQR]^i^								
Male Ref: 8.3–10.5		6.8 [5.7–7.7]	8.1 [6.9–8.7]	8.7 [8.2–9.0]	9.3 [8.9–9.8]	<0.05	<0.05	
Female Ref: 7.3–9.5		5.4 [4.8–6.7]	7.8 [6.4–8.3]	8.3 [7.6–8.7]	8.6 [8.5–8.8]			
WBC *10^9^/L [IQR] Ref: 3.50–8.80		10.4 [8.4–14.7]	6.9 [6.3–9.8]	6.3 [4.9–10.2]	6.1 [5.8–6.6]	<0.05		
Neutrophils *10^9^/L [IQR] Ref: 1.50–7.50		7.9 [5.3–9.6]	4.5 [3.4–6.2]	3.8 [2.4–6.6]	3.3 [2.6–4.0]	<0.05		
Monocytes *10^9^/L [IQR] Ref: 0.20–0.80		0.8 [0.6–1.6]	0.6 [0.5–1.1]	0.4 [0.4–0.7]	0.5 [0.4–0.5]			
Median cytokine [IQR] in pg/ml								
**Null**	IFN-γ	12 [9.7–19]	26 [16–64]	10 [4.7–13]	17 [8.7–39]		<0.05	
	IL-1β	0.7 [0.1–1.7]	1.2 [0.2–5.8]	0.2 [0.1–0.5]	0.9 [0.4–6.4]		<0.05	<0.05
	IL-2	0.4 [0.3–2.2]	0.4 [0.1–2.3]	0.4 [0.1–0.6]	0.6 [0.4–0.9]			
	IL-4	0.4 [0.1–1.0]	0.3 [0.1–0.6]	0.2 [0.0–0.2]	0.3 [0.2–0.5]			<0.01
	IL-6	2.7 [1.4–4.8]	3.0 [0.8–7.4]	0.5 [0.4–1.5]	1.4 [0.7–3.0]		<0.01	<0.05
	IL-8	32 [9–360]	193 [33–683]	14 [10–18]	30 [14–113]		<0.001	
	IL-10	0.9 [0.0–3.0]	0.4 [0.2–1.6]	0.2 [0.1–0.3]	0.5 [0.3–1.7]		<0.01	<0.05
	IL-12p70	0.3 [0.0–0.7]	0.5 [0.2–1.8]	0.2 [0.0–1.0]	0.5 [0.2–1.0]			
	IL-13	7.7 [3.1–8.3]	6.5 [2.4–13]	2.0 [1.5–2.6]	3.4 [2.2–10]		<0.01	
	TNF-α	3.2 [1.9–4.7]	4.7 [2.2–23]	1.2 [0.7–3.2]	2.3 [1.4–11]		<0.01	
**Anti-CD** **3 and 28**	IFN-γ	740 [397–3,791]	3,395 [1,193–10,489]	652 [70–5,254]	4,557 [559–13,192]			
	IL-1β	10 [1.8–41]	23 [8.8–63]	1.8 [1.2–4.5]	19 [6.2–21]		<0.01	<0.05
	IL-2	114 [50–314]	241 [88–483]	62 [4.4–241]	252 [65–510]			
	IL-4	1.9 [0.7–10]	9.3 [4.0–23]	1.2 [0.1–3.7]	11 [2.1–22]		<0.05	
	IL-6	7.8 [2.1–23]	20 [8.0–50]	1.8 [1.4–6.3]	15 [7.3–24]		<0.01	<0.05
	IL-8	1,808 [369–1,971]	1,784 [1,159–2,423]	188 [98–564]	1,104 [400–1,793]		<0.001	<0.05
	IL-10	4.9 [3.2–38]	35 [8.7–61]	11 [0.5–35]	76 [29–115]			
	IL-12p70	1.8 [0.8–2.7]	2.2 [0.6–4.3]	0.7 [0.3–0.9]	1.4 [1.1–4.1]		<0.05	<0.01
	IL-13	42 [12–50]	45.9 [24.1–70]	7.1 [4.6–12]	31 [11–44]		<0.001	<0.05
	TNF-α	34 [25–243]	264 [67.8–580]	42 [7.1–305]	473 [74–772]			<0.05
**LPS (TLR4)**	IFN-γ	61 [41–456]	1,397 [581–4,177]	3,043 [2,127–3,655]	3,724 [1,027–8,051]	<0.05		
	IL-1β	1,297 [176–1,998]	2,094 [1,076–3,025]	1,543 [1,174–2,178]	2,117 [1,813–2,813]			
	IL-2	15 [13–24]	18 [13.1–29]	13 [9.3–22]	22 [16–26]			
	IL-4	53 [12–83]	25 [20–45]	51 [20–63]	62 [41–66]			
	IL-6	4,075 [1,379–7,241]	5,429 [4,759–7,674]	5,555 [4,618–7,169]	5,151 [4,658–5,667]			
	IL-8	4,449 [2,271–5,038]	4,421 [4,209–6,391]	4,831 [4,391–6,519]	4,254 [3,598–6,474]			
	IL-10	44 [19–129]	71 [29–109]	93 [71–202]	131 [104–141]			
	IL-12p70	38 [26–103]	46 [28–109]	65 [32–120]	93 [66–110]			
	IL-13	131 [84–138]	97 [83–124]	87 [78–117]	135 [118–157]			<0.01
	TNF-α	888 [451–2,599]	1,931 [1,162–2 602]	1,695 [1,181–3,024]	1,741 [1,283–2,210]			
**ZYM (TLR2)**	IFN-γ	55 [33–351]	1,401 [408–2,677]	2,868 [1,738–5,637]	4,687 [2,052–16,369]	<0.05		
	IL-1β	1,082 [129–1,657]	1,881 [1,310–2,002]	1,120 [1,098–1,587]	1,796 [1,503–1,970]	<0.05	<0.01	<0.05
	IL-2	11 [8.3–15]	16 [11–31]	11 [7.1–24]	20 [14–24]			
	IL-4	29 [5.2–34]	20 [13–34]	20 [14–42]	34 [27–52]			
	IL-6	2,635 [519–3,871]	2,599 [2,482–3,976]	3,795 [2,433–3,891]	3,943 [2,819–4,564]			
	IL-8	2,373 [2,188–2,410]	2,251 [2,112–3,145]	2,414 [2,224–3,041]	3,074 [2,056–3,140]			
	IL-10	39 [19–116]	144 [70–207]	83 [61–172]	150 [99–196]	<0.05		
	IL-12p70	54 [8–73]	39 [28–61]	38 [22–56]	44 [38–74]			
	IL-13	75 [45–92]	83 [61–99]	70 [53–97]	73 [63–102]			
	TNF-α	995 [487–3,506]	4,011 [2,972–4,720]	3,164 [1,416–4,333]	2,730 [2,588–4,678]	<0.05		

n = 52. Imputed values included except for hemoglobin due to min–max scaling. ^i^Without imputed values. Anti-CD3 and 28, anti-cluster of differentiations 3 and 28; CMTB, conditions mimicking tuberculosis; CRP, C-reactive protein; IFN-γ, interferon-gamma; HC, healthy controls; IL, interleukin; LPS, lipopolysaccharides; IQR, interquartile range; Ref, laboratory reference ranges; TNF-α, tumor necrosis factor α; TLR, Toll-like receptor; WBC, total white blood cell count; TB, tuberculosis; TBI, tuberculosis infection; ZYM, zymosan.

### Biomarker combinations to differentiate between TB and CMTB

**Figure 6:**
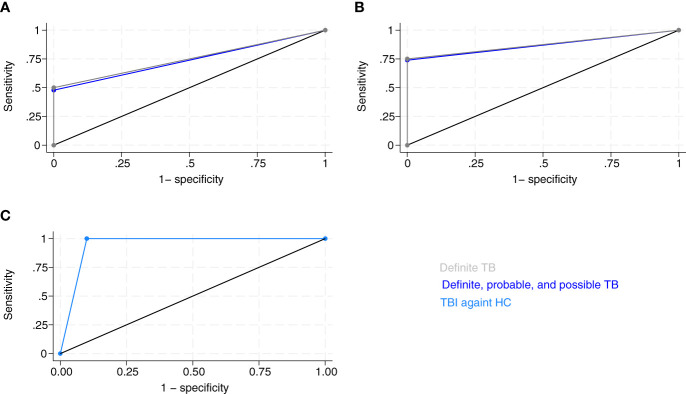
Receiver operating curves (ROC) of index test by groups. **(A)** ROC curve of neutrophils, LPS-IFN-γ, ZYM-IFN-γ, ZYM-TNF-α, ZYM-IL-1β, LPS-IL-4, and ZYM-IL-6 discriminated tuberculosis (TB) and conditions mimicking tuberculosis (CMTB), and definite TB from CMTB. Area under ROC curve of definite, probable, and possible TB against CMTB = 0.76 (95% CI: 0.655–0.867), n = 32 (dark blue). Sub-group analysis of definite TB against CMTB: area under ROC curve = 0.792 (95% CI: 0.653–0.931), n = 21 (gray). **(B)** ROC curve of Null-IFN-γ, Null-IL-8, CD3-IL-6, CD3-IL-8, CD3-IL-13, and ZYM-IL-1β for differentiation of TB and tuberculosis infection (TBI). Area under ROC curve of definite, probable, and possible TB against TBI = 0.87 (95% CI: 0.779–0.96), n = 33 (dark blue). Sub-group analysis of definite TB against TBI: area under ROC curve = 0.875 (95% CI: 0.756–0.993), n = 22 (gray). **(C)** ROC curve of Null-IL-1β, Null-IL-4, CD3-IL-1β, CD3-IL-12p70, and LPS-IL-13 for differentiation of tuberculosis infection (TBI) and healthy controls (HC). Area under ROC curve = 0.95 (95% CI: 0.854–1.05), n = 20 (light blue).

We identified the most potent biomarkers by recursive feature elimination in each group (**Supplementary Figure S2**) and included these in index tests to discriminate the four groups (**Supplementary Table S2**). We selected cut-off values of biomarkers for testing positive for TB to fit the criteria of ≥98% specificity (**Table 3**). Neutrophils, LPS-IFN-γ, ZYM-IFN-γ, ZYM-TNF-α, ZYM-IL-1β, LPS-IL-4, and LPS-IL-6 discriminated TB from CMTB with a sensitivity (95% CI) of 52.2% (30.9%–73.4%) and specificity of 100% (95% CI: 66.4%-100%). The area under the curve (AUC) was 0.76 (**Figure 6A**). We performed a subgroup analysis of patients with definite TB (n = 12) compared to CMTB using the same parameters and cut-offs. Sensitivity was then 58.3% (21.2%–78.8%), specificity was 100% (66.4%–100%), and the AUC was 0.792 (0.651–0.933).

**Table 3:** Index test variables and cut-offs for tuberculosis against conditions mimicking tuberculosis and subgroup analysis, including only definite tuberculosis and tuberculosis against tuberculosis infection, and subgroup analysis, including only definite tuberculosis and tuberculosis against healthy controls.

Group	n	Index test variables	Selected cut-off values for index test positive for TB:	Sensitivity (95% CIs)	Specificity (95% CIs)	AUC (95% CIs)
TB vs. CMTB	32	Neutrophils	<5.3 *10^9^/L	47.8% (26.8–67.7)	100% (66.4–100)	0.740 (0.640–0.840)
		ZYM-IL1b	≥1,000 pg/ml			
		ZYM-IFN-γ	≥100 pg/ml			
		ZYM-TNF-α	≥900 pg/ml			
		LPS-IL-4	<50 pg/ml			
		LPS-IL-10	>25 pg/ml			
		LPS-IFN-γ	≥200 pg/ml			
Subgroup analysis: Definite TB vs. CMTB	21	Neutrophils	<5.3 *10^9^/L	58.3% (21.2–78.8)	100% (66.4–100)	0.792 (0.651–0.933)
		ZYM IL-1β	≥1,000 pg/ml			
		ZYM-IFN-γ	≥100 pg/ml			
		ZYM-TNF-α	≥900 pg/ml			
		LPS-IL-4	<50 pg/ml			
		LPS-IL-10	>25 pg/ml			
		LPS-IFN-γ	≥200 pg/ml			
TB vs. TBI	33	Null IFN-y	>5 pg/ml	73.9% (56.5–91.3)	100% (69.2–100.0)	0.870 (0.780–0.960)
		Null IL-8	>30 pg/ml			
		CD3 IL-6	>4 pg/ml			
		CD3 IL-8	>600 pg/ml			
		CD3 IL-13	>10 pg/ml			
		ZYM IL-1β	>1,150 pg/ml			
Subgroup analysis: Definite TB vs. TBI	22	Null IFN-y	>5 pg/ml	75.0% (49.8–100.2)	100% (69.2–100)	0.875 (0.756–0.993)
		Null IL-8	>30 pg/ml			
		CD3 IL-6	>4 pg/ml			
		CD3 IL-8	>600 pg/ml			
		CD3 IL-13	>10 pg/ml			
		ZYM IL-1β	>1,150 pg/ml			
		Null IFN-y	>5 pg/ml			
TBI vs. HC	20	Null-IL-1β	<1.0 pg/ml	100% (69.2–100)	90.0% (70.2–109)	0.950 (0.854–1.05
		Null-IL-4	<1.0 pg/ml			
		CD3-IL-1β	<50 pg/ml			
		CD3-IL-12p70	<4 pg/ml			
		LPS-IL-13	<133 pg/ml			

AUC, area under the curve; CD3, anti-cluster of differentiations 3 and 28 stimulated; CMTB, tuberculosis-mimicking disease; HC, healthy controls; IFN-γ, interferon-gamma; IL, interleukin; LPS, lipopolysaccharides stimulated; TNF-α, tumor necrosis factor α; TB, tuberculosis; TBI, tuberculosis infection; ZYM, zymosan stimulated.

### Biomarker combinations to differentiate between TB and TBI

Null-IFN-γ, Null-IL-8, CD3-IL-6, CD3-IL-8, CD3-IL-13, and ZYM IL-1β discriminated TB from TBI with a sensitivity of 73.9% (56.5%–91.3%) and a specificity of 100% (69.2%–100%). The AUC was 0.870 (0.779–0.960) (**Figure 6B**). In a subgroup analysis of patients with definite TB (n = 12) compared to TBI using the same parameters and cut-offs, sensitivity was 75.0% (49.8%–100.2%), specificity was 100% (69.2%–100%), and AUC was 0.875 (0.756–0.993).

### Biomarker combinations to differentiate between TBI and HC

Null-IL1β, Null-IL-4, CD3-IL-1β, CD3-IL-12p70, and LPS-IL-13 differentiated TBI from HC with a sensitivity of 100% (69.2%–100%), specificity of 90.0% (70.2%–109%), and AUC of 0.950 (0.854–1.05) (**Figure 6C**).

## Discussion

We investigated the expression of 10 cytokines, hemoglobin, CRP, WBC, neutrophils, and monocytes after *ex vivo* stimulation of the innate and adaptive immune systems in patients with TB, TBI, CMTB, and HCs. We tested the ability of these biomarkers to differentiate TB from CMTB and TBI to achieve the minimum diagnostic accuracy specified in the WHO target product profile, which requires a specificity of ≥98%. We found that neutrophil counts combined with six cytokines (LPS-IFN-γ, ZYM-IFN-γ, ZYM-TNF-α, ZYM-IL-1β, LPS-IL-4, and ZYM-IL-6) differentiated TB from CMTB with a sensitivity of 52.2% and a specificity of 100%. The study also found that a test consisting of six cytokines (Null-IFN-γ, Null-IL-8, CD3-IL-6, CD3-IL-8, CD3-IL-13, and ZYM IL-1β) discriminated TB from TBI with a sensitivity of 73.9% and a specificity of 100%. Finally, we found no combined test to discriminate TBI from HC with the desirable specificity.

Of previously established biomarkers for infection and inflammation, we found that CRP, WBC, and neutrophil counts increased in TB and CMTB compared to TBI and HC. Meyer et al. evaluated the diagnostic performance of CRP in diagnosing TB in Uganda, a highly endemic country, in 119 patients referred for TB evaluation (34). Using a 10-mg/L cut-off, they found a sensitivity of 78% and a specificity of 52%. In contrast, our data showed higher levels of CRP in CMTB. These findings depend on the patients included in CMTB. Meyer et al. did not provide specific diagnoses of the persons not diagnosed with TB for further comparison. The levels of hemoglobin and WBC in the TB group and HC were consistent with previous findings (35). We interpret no significant difference between definite and probable/possible TB in any of the 45 biomarkers, and the findings similar specificity, sensitivity, and AUCs of the subgroup analysis of definite TB as a low dependency of bacterial load in cytokine response. Previously, no association has been demonstrated between the bacterial burden in culture-positive PTB and unstimulated Luminex-analyzed plasma levels of IL-1β, IL-4, IL5, IL-6, IL-10, and IL-12 (36). However, increasing plasma levels of unstimulated Bio-Plex-analyzed IFN-γ and TNF-α were observed in culture-positive PTB with increasing microscopy smear-grade positivity (37).

This study is unique in relying on innate and adaptive immune stimulation to delineate cytokine responses in TB, CMTB, TBI and HC, suggesting the benefit of incorporating both immune arms in these distinctions. TB patients were characterized by high levels of innate immune system-stimulated cytokines in TB compared to CMTB, which substantiates the importance of both TLR2 and TLR4 in the immune detection of *Mtb* (25, 26). In addition, T-cell-derived and spontaneously released cytokines helped differentiate TB from TBI, likely reflecting the greater degree of *Mtb* immune priming in TB compared to TBI. Cytokine differences in unstimulated tubes were generally minor in the current study, and we found no significant differences between TB and CMTB. This finding indicates that stimulation of cytokines is a valuable tool for differentiating TB from CMTB.

Diagnosis of TB remains challenging despite recent developments in sputum-based diagnostics (38). The elevated levels of IL-12, IL-6, and TNF-α after ZYM stimulation is in line with previously described results (39). Other studies have investigated unstimulated or *Mtb*-antigen-stimulated cytokines. Ren et al. analyzed 12 pro-inflammatory cytokines in sera of patients with PTB, patients co-infected with TB and chronic pulmonary aspergillosis (CPA), CPA patients with tumors or pneumonia (CPA+), and HC. The study likewise found lower WBC and neutrophil counts in TB patients compared to both CPA+ and co-infected TB and CPA (22). The study examined combinations of IL-1β and IL-8, IL-8 and TNF-α, and IL-1β, IL-8, and TNF-α based on significant p-values to discriminate between TB and CPA+. Interestingly, an AUC of ≥0.974 in all combinations was reported. These findings support our utilization of neutrophil counts combined with IFN-γ, TNF-α, and IL-1β cytokine levels to differentiate TB from CMTB and PTB from EPTB, as well as to a different non-TB disease group.

TruCulture-derived cytokine profiles have been evaluated against QuantiFERON*^®^*-TB Gold in tube after *Mtb* stimulation in persons with TB and TBI (40). The study found a significant difference in stimulated IFN-γ response between TBI and TB in the TruCulture assay but not in the QuantiFERON*^®^*-TB Gold in tube, suggesting that the TruCulture assay may be more sensitive.

In the current setup, the analysis of cytokines is expensive and requires advanced laboratory facilities. Assays have been produced for point-of-care testing (41), and TLR4-stimulated cytokine levels have been stimulated in whole blood for a shorter time (4 h) (42) suggesting that cytokine assays could be produced as a point-of-care test with minimum handling and short turn-around time, which is required to have clinical relevance in a high-TB incidence rate low-resource setting. The combined cytokine test have potential as a non-invasive add-on test to established TB diagnostics to detect TB in patients suspected of TB. It is essential to validate these results in larger patient populations and settings with higher TB incidence rate.

The IFN-γ response specific to *Mtb* is the basis for TBI diagnostics in IGRAs (43), but IGRAs cannot differentiate between TB, TBI, and CMTB with positive IGRA results. Our findings demonstrate the importance of IFN-y in innate immunity to discriminate between TB and CMTB when stimulated by ZYM and LPS and between TB and TBI. This new combined cytokine test shows the potential in discriminating TBI from TB.

The study strengths include the broad group of persons with CMTB suspected of TB and a broad group of TB patients, which makes the test clinically relevant as a guidance in diagnostics for initiation of antituberculous treatment. Second, including all of probable, possible, and definite TB allows for the combined cytokine tests in persons with paucibacillary TB, as the subgroup analysis of definite TB cases showed similar sensitivity and specificity. For different TB subgroups, the use of standardized adaptive and innate immune stimulation, the latter including the *Mtb* relevant TLR2 and TLR4 receptors, in conjunction with a standardized cytokine panel, enabled us to delineate adaptive and innate immune sensing mimicking *in vivo* sensing of *Mtb*.

The main limitations of this study include the small sample size, broad confidence intervals of sensitivity and specificity, and a lack of validation in settings with high TB incidence rates in immunosuppressed individuals, such as persons living with HIV or inflammatory diseases, and in children. The study sample size and selection of cut-offs after data collection may result in overly optimistic test estimates. Nonetheless, the combinations of IL-1β, IL-8, and TNF-α to discriminate between PTB and CMTB were found in a different setting, using another analysis method, and chosen by p-values (22), in contrast to the recursive feature elimination using a random forest model we used to select cytokines, which support the validation of the findings. Further, different assays have previously shown varying sensitivity and specificity (44), and there is currently no reference standardization of cytokine measurements. We did not measure the WBC in the TruCulture tubes, which could implicate the cytokine production during stimulation with more cytokine production with higher WBC that was present in CMTB.

A limitation of the found index test that discriminates between TB and CMTB is TNF-α inclusion, which may limit its value in patients receiving TNF-α inhibitor therapy. It could be clinically relevant to distinguish between the progression of inflammatory bowel symptoms and TB, and validating these findings should include this patient group.

Cytokines as biomarkers have challenges regarding low trace amounts, short half-lives, complex networks of up- and downregulation in responses to the microcellular milieu and changed levels with repeated thawing (45). We studied *ex vivo* whole-blood cytokines using the highly sensitive and antigen concentration standardized TruCulture assay, which requires minimal blood volume and technical experience to perform (46). We handled the samples within the same short time frame to reduce bias and thawed the samples no more than three times minimizing the risk of changed cytokine levels. We assessed this in a few samples and used the first analysis of the sample to avoid change in cytokine level.

## Conclusion

This explorative study suggests that combinations of six stimulated whole-blood cytokines using TruCulture tubes and established biomarkers can differentiate TB from CMTB, and four stimulated and two unstimulated cytokines can differentiate TB and TBI with a high specificity and acceptable sensitivity. This may serve as a promising non-invasive blood-based add-on test in TB diagnostics in adult patients. The sample size was small, and the findings require broader validation including also immunosuppressed patients and children.

## Data availability statement

The datasets presented in this article are not readily available because of General Data Protection Regulations; https://www.bbmri-eric.eu/wp-content/uploads/BBMRI-ERIC_FAQs_on_the_GDPR_V2.0-1.pdf. Requests to access the datasets should be directed to anne.ahrens.ostergaard@rsyd.dk.

## Ethics statement

The studies involving humans were approved by Danish National Committee of Health Research Ethics. The studies were conducted in accordance with the local legislation and institutional requirements. The participants provided their written informed consent to participate in this study.

## Author contributions

AA: Writing – original draft, Writing – review & editing. SF: Writing – review & editing. MBa: Writing – review & editing. RL: Writing – review & editing. AK: Writing – review & editing. MBo: Writing – review & editing. IT: Writing – review & editing. TJ: Writing – review & editing. OH: Writing – review & editing. CW: Writing – review & editing. SB: Writing – review & editing. MBl: Writing – review & editing. KA: Writing – original draft, Writing – review & editing. IJ: Writing – original draft, Writing – review & editing.
