# Supplementary material for: Whole-blood culture-derived cytokine combinations for the diagnosis of tuberculosis
Source: Front Immunol. 2024 Jun 12;15:1397941. doi: 10.3389/fimmu.2024.1397941 (PMC11199390; doi:10.3389/fimmu.2024.1397941)
Supplement: Supplementary file 1 [file DataSheet_1.docx]

Supplementary Material

# Supplementary materials

## Cytokine analysis:

We analysed cytokines in three runs. Samples were diluted 2- or 4-fold in the first and second run. In the third run, we re-ran samples with a coefficient of variation (CV) >25 and samples above the detection range /upper limit of detection (ULOD) in either 4- or 8-fold dilution, depending on previous results. We excluded values with CV>25 after re-analysis (4 samples). We ran eight samples from three patients in all runs to determine the inter-analysis variation. We included the earliest analysis and the lowest dilution within the detection range in case of multiple determinations. No personnel assessing cytokines analysis were blinded to other participant characteristics and other laboratory results.

After a re-run of cytokine levels in higher dilutions, 276 (13.6%) cytokine measurements were above the upper limit of detection (ULOD). Meso Scale Discovery platform estimates a value of cytokine level by four-parameter logistic regression for ULOD measurements (imputation from a fitted distribution). We included these values in the analysis. For cytokine levels below the lower limit of detection (LLOD) (n=51, 2.5%), a value of 0.5 LLOD was included. For CPR levels below LLOD of 0.6mg/L, a value of 0.5 LLOD=0.3mg/L was included.

# Supplementary Tables

## Table S1.

Biomarkers in tuberculosis, tuberculosis infection, conditions mimicking tuberculosis, and healthy controls, n=52. Exclusive imputed values

| **Variables** | | **CMTB** | **TB** | **TBI** | **HC** | **Significant differences in groups,**  **p-value^i^** | | |
| --- | --- | --- | --- | --- | --- | --- | --- | --- |
| **n (%)** | | 9 (17.3) | 23 (44.2) | 10 (19.2) | 10 (19.2) | **TB vs CMTB** | **TB vs TBI** | **TBI vs HC** |
| **CRP mg/L [IQR]**  ref:<5.0 | | 31.5  [6.9-58.0] (n=8) | 7.1  [2.5-23.0] (n=21) | 0.9  [0.3-2.0] (n=9) | 0.8  [0.6-1.4] (n=10) |  | <0.05 |  |
| **Hemoglobin mmol/L [IQR]** | | | | | | | | |
| Male  Ref: 8.3-10.5 | | 6.8  [5.7-7.7] (n=8) | 8.1  [6.9-8.7] (n=20) | 8.7  [8.2-9.0] (n=9) | 9.3  [8.9-9.8] (n=4) | <0.05 | <0.05 |  |
| Female  Ref:7.3-9.5 | | 5.4  [4.8-6.7] (n=3) | 7.8  [6.4-8.3] (n=10) | 8.3  [7.6-8.7] (n=6) | 8.6  [8.5-8.8] (n=4) |  |  |  |
| **Total leucocytes *10^9^/L [IQR]**  Ref:3.50-8.80 | | 11.2  [8.0-18.7] (n=8) | 6.8  [6.3-8.8] (n=20) | 6.3  [4.9-10.2] (n=10) | 6.1  [5.8-6.6] (n=10) | <0.05 |  |  |
| Neutrophils *10^9^/L [IQR]  Ref**:**1.50-7.50 | | 8.5  [5.6-13.9] (n=8) | 4.1  [3.4-6.1] (n=19) | 3.8  [2.4-6.6] (n=10) | 3.3  [2.6-3.8] (n=9) | <0.05 |  |  |
| Monocytes *10^9^/L [IQR]  Ref:0.20-0.80 | | 1.2  [0.6-1.7] (n=8) | 0.5  [0.4-0.9] (n=14) | 0.4  [0.4-0.7] (n=9) | 0.4  [0.4-0.5] (n=8) |  |  |  |
| **Median cytokine [IQR] in pg/mL** | | | | | | | | |
| **Null /**  **Un-stimulated** | IFN-γ | 13 [5.0-60] | 26 [16-64] | 10 [4.7-13] | 17 [8.7-39] |  | <0.05 |  |
|  | IL-1β | 0.5 [0.1-1.9] | 1.2 [0.2-5.8] | 0.2  [0.1-0.5] | 0.9 [0.4-6.4] |  | <0.05 | <0.05 |
|  | IL-2 | 1.1 [0.2-2.5] | 0.4 [0.1-2.3] | 0.4 [0.1-0.6] | 0.6 [0.4-0.9] |  |  |  |
|  | IL-4 | 0.4 [0.0-1.1] | 0.3 [0.1-0.6] | 0.2 [0.0-0.2] | 0.3 [0.2-0.5] |  |  | <0.01 |
|  | IL-6 | 2.9 [1.0-6.3] | 3.0 [0.8-7.4] | 0.5 [0.4-1.5] | 1.4 [0.7-3.0] |  | <0.01 | <0.05 |
|  | IL-8 | 24 [ 7-327] | 193 [33-683] | 14 [10-18] | 30 [14-113] |  | <0.001 |  |
|  | IL-10 | 0.7 [0.0-3.3] | 0.4 [0.2-1.6] | 0.2 [0.1-0.3] | 0.5 [0.3-1.7] |  | <0.01 | <0.05 |
|  | IL-12p70 | 0.2 [0.0-1.2] | 0.5 [0.2-1.8] | 0.2 [0.0-1.0] | 0.5 [0.2-1.0] |  |  |  |
|  | IL-13 | 6.3 [2.1-9.3] | 6.5 [2.4-13] | 2.0 [1.5-2.6] | 3.4 [2.2-10] |  | <0.01 |  |
|  | TNF-α | 2.6 [1.2-4.0] | 4.7 [2.2-23] | 1.2 [0.7-3.2] | 2.3 [1.4-11] |  | <0.01 |  |
| **Anti-CD3/CD28**  **stimulated** | IFN-γ | 740 [128-3791] | 3395 [1193-9347] | 652 [ 70-5254] | 4557 [559-13192] |  |  |  |
|  | IL-1β | 10 [1.8-41] | 23 [8.8-60] | 1.8 [1.2-4.5] | 19 [6.2-21] |  | <0.01 | <0.05 |
|  | IL-2 | 114 [38-437] | 239 [ 88-416] | 62 [4.4-241] | 252 [65-510] |  |  |  |
|  | IL-4 | 1.9 [0.7-24] | 9.2 [4.0-17] | 1.2 [0.1-3.7] | 11 [2.1-22] |  | <0.05 |  |
|  | IL-6 | 7.8 [2.1-85] | 20 [8.0-42] | 1.8 [1.4-6.3] | 15 [7.3-24] |  | <0.01 | <0.05 |
|  | IL-8 | 1808 [369-2019] | 1784 [1159-2297] | 188 [ 98-564] | 1104 [400-1793] |  | <0.001 | <0.05 |
|  | IL-10 | 4.9 [3.2-120] | 30 [8.7-53] | 11 [0.5-35] | 76 [ 29-115] |  |  |  |
|  | IL-12p70 | 1.8 [0.8-9.8] | 2.2 [0.6-3.3] | 0.7 [0.3-0.9] | 1.4 [1.1-4.1] |  | <0.05 | <0.01 |
|  | IL-13 | 42 [ 12-134] | 38.7 [24.1-69] | 7.1 [4.6-12] | 31 [11-44] |  | <0.001 | <0.05 |
|  | TNF-α | 34 [ 25-330] | 248 [67.8-506] | 42 [7-305] | 473 [ 74-772] |  |  | <0.05 |
| **LPS**  **stimulated** | IFN-γ | 61 [ 41-456] | 1397 [581-4177] | 3043 [2127-3655] | 3724 [1027-8051] | <0.05 |  |  |
|  | IL-1β | 1297 [176-1998] | 2094 [1076-3025] | 1543 [1174-2178] | 2117 [1813-2813] |  |  |  |
|  | IL-2 | 15 [ 13-24] | 18 [13.1-29] | 13 [9.3-22] | 22 [16-26] |  |  |  |
|  | IL-4 | 53 [12-83] | 25 [20-45] | 51 [20-63] | 62 [41-66] |  |  |  |
|  | IL-6 | 4075 [1379-7241] | 5429 [4759-7674] | 5555 [4618-7169] | 5151 [4658-5667] |  |  |  |
|  | IL-8 | 4449 [2271-5038] | 4421 [4209-6391] | 4831 [4391-6519] | 4254 [3598-6474] |  |  |  |
|  | IL-10 | 44 [19-129] | 71 [29-109] | 93 [81-202] | 131 [104-141] |  |  |  |
|  | IL-12p70 | 38 [26-103] | 46 [28-109] | 65 [32-120] | 93 [66-110] |  |  |  |
|  | IL-13 | 131 [84-138] | 97 [83-124] | 87 [78-117] | 135 [118-157] |  |  | <0.01 |
|  | TNF-α | 888 [451-2599] | 1931 [1162-2602] | 1695 [1181-3024] | 1741 [1283-2210] |  |  |  |
| **ZYM**  **stimulated** | IFN-γ | 55 [33-351] | 1401 [408-2677] | 2868 [1738-5637] | 4687 [2052-16369] | <0.05 |  |  |
|  | IL-1β | 1082 [129-1657] | 1881 [1310-2002] | 1120 [1098-1587] | 1796 [1503-1970] | <0.05 | <0.01 | <0.05 |
|  | IL-2 | 11 [8.3-15] | 16 [11-31] | 11 [7.1-24] | 20 [ 14-24] |  |  |  |
|  | IL-4 | 29 [5.2-34] | 20 [13-34] | 20 [14-42] | 34 [27-52] |  |  |  |
|  | IL-6 | 2635 [519-3871] | 2599 [2482-3976] | 3795 [2433-3891] | 3943 [2819-4564] |  |  |  |
|  | IL-8 | 2373 [2188-2410] | 2251 [2112-3145] | 2414 [2224-3041] | 3074 [2056-3140] |  |  |  |
|  | IL-10 | 39 [19-116] | 144 [70-207] | 83 [61-172] | 150 [99-196] | <0.05 |  |  |
|  | IL-12p70 | 54 [8-73] | 39 [ 28-61] | 38 [22-56] | 44 [38-74] |  |  |  |
|  | IL-13 | 75 [45-92] | 83 [61-99] | 70 [53-97] | 73 [63-102] |  |  |  |
|  | TNF-α | 995 [487-3506] | 4011 [2972-4720] | 3164 [1416-4333] | 2730 [2588-4678] | <0.05 |  |  |

^i^ inclusive imputed values. Anti-CD3/CD28: Anti-cluster of differentiations 3 and 28. CMTB: Tuberculosis-mimicking disease. CRP: C-reactive protein. HC: Healthy controls. IFN-ɣ: Interferon-gamma. IL: Interleukin. IQR: Interquartile range. LPS: Lipopolysaccharides. Ref: Laboratory reference ranges. TNF-α: Tumour necrosis Factor α. TB: Tuberculosis. TBI: Tuberculosis infection. ZYM: Zymosan

# Supplementary Figures

## Figure S1.

Logarithmic mean concentration of cytokines of eight participants’ supernatants in four tubes (CD3, LPS, Null and ZYM) analysed in three different runs (1-3). As the symbols overlay, there was limited inter-analysis variation. Anti-CD3/CD28: Anti-cluster of differentiations 3 and 28. IFN-ɣ: Interferon-gamma. IL: Interleukin. LPS: Lipopolysaccharides. TNF-α: Tumour necrosis Factor α. ZYM: Zymosan

.

## Figure S2

Random forest feature importance from recursive feature elimination selected biomarkers in **A**: Tuberculosis vs conditions mimicking tuberculosis, **B**: Tuberculosis infection vs tuberculosis **C**: Tuberculosis infection vs healthy controls.

CD3: Stimulated with anti- cluster of differentiations (CD) 3 and 28. IFN-ɣ: Interferon-gamma. IL: Interleukin. LPS: Lipopolysaccharides (toll-like receptor (TLR) 4). Null: Unstimulated. TNF-α: Tumour necrosis Factor α. ZYM: Zymosan (TLR2).

**A**:

Importance

LPS-IL-6

ZYM-IL-1β

LPS-IL-4

ZYM-TNF-α

ZYM-IFN-ɣ

LPS-IFN-ɣ


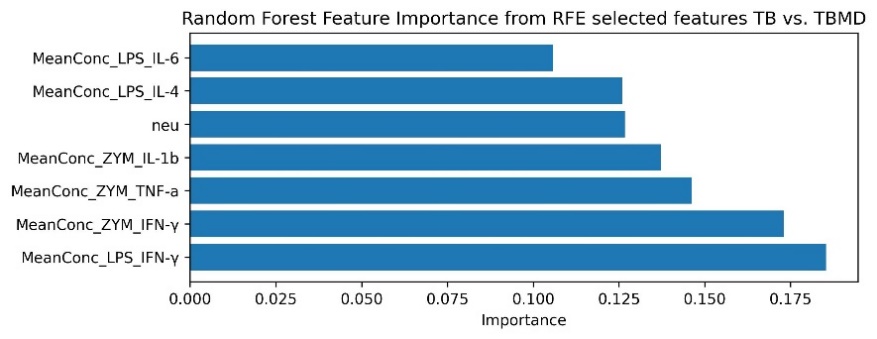


Neutrophil counts

0.000

0.025

0.050

0.075

0.100

0.125

0.150

0.175

**B:**


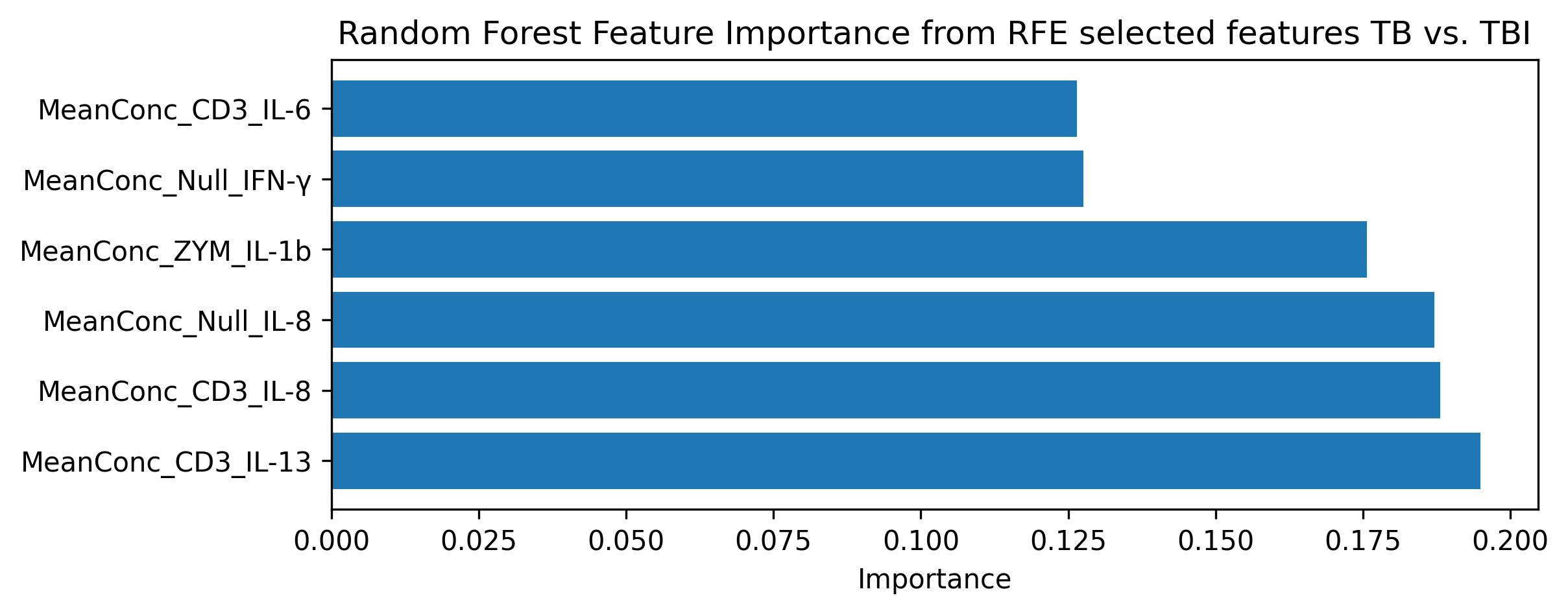


0.000

0.025

0.050

0.075

0.100

0.125

0.150

0.175

Importance

0.200

CD3-IL-8

CD3-IL-13

ZYM-IL-1β

CD3-IL-6

Null-IL-8

Null-IFN-ɣ

**C:**


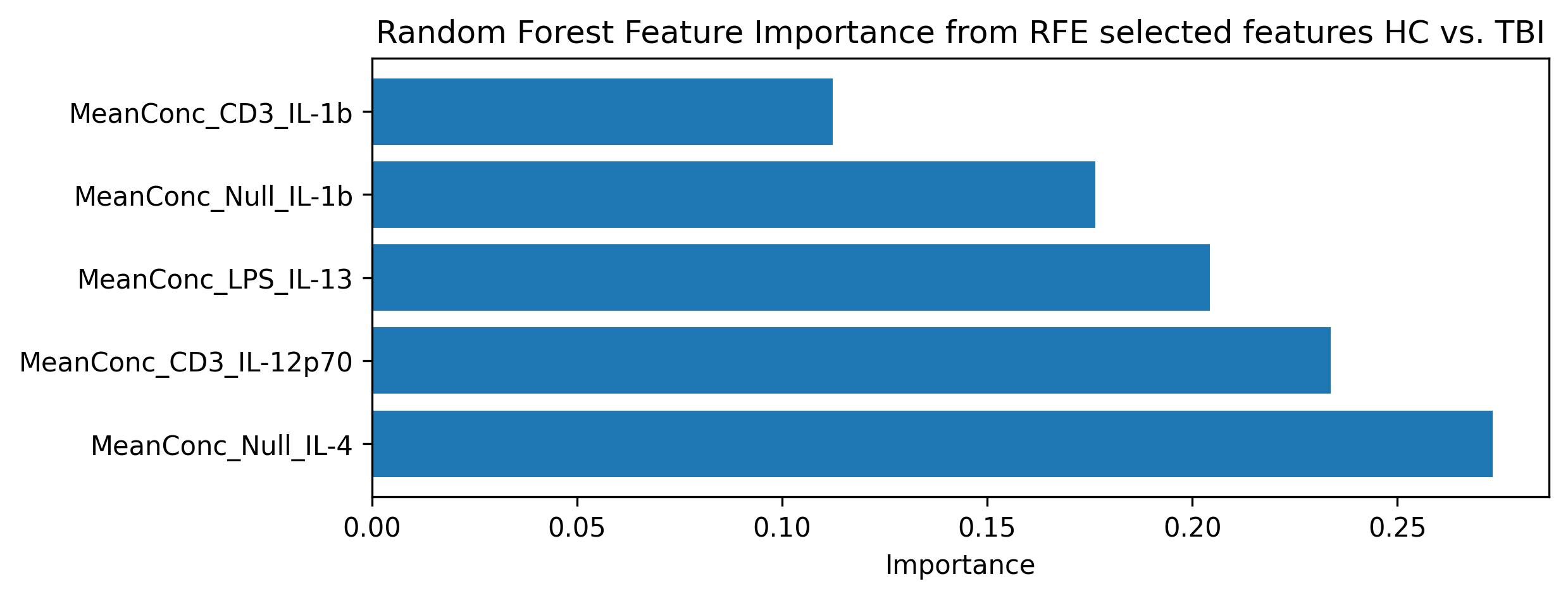


Null-IL-4

CD3-IL-1β

NUll-IL-1β

CD3-IL-12p70

LPS-IL-13

0.000

0.050

0.10

0.15

0.20

0.25

Importance

**
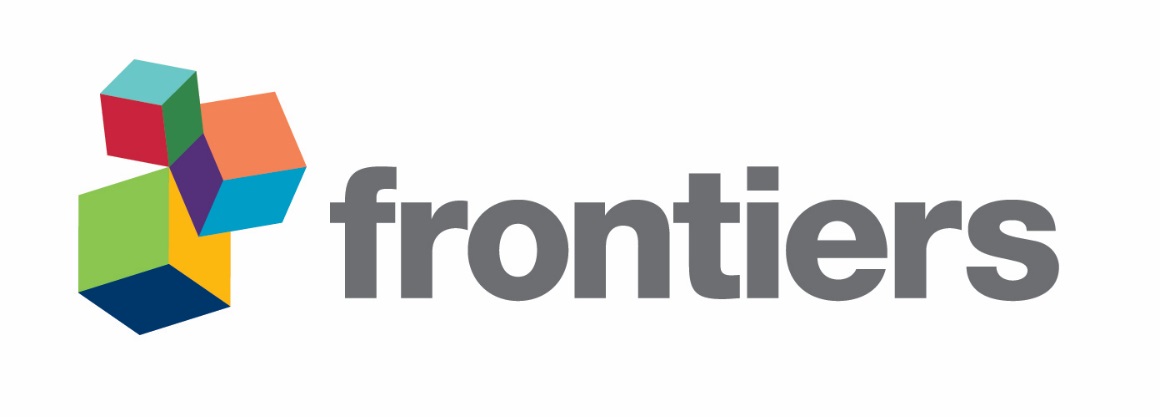
**
